# High human papillomavirus (HPV) prevalence in South African adolescents and young women encourages expanded HPV vaccination campaigns

**DOI:** 10.1371/journal.pone.0190166

**Published:** 2018-01-02

**Authors:** Zizipho Z. A. Mbulawa, Cari van Schalkwyk, Nai-Chung Hu, Tracy L. Meiring, Shaun Barnabas, Smritee Dabee, Heather Jaspan, Jean-Mari Kriek, Shameem Z. Jaumdally, Etienne Muller, Linda-Gail Bekker, David A. Lewis, Janan Dietrich, Glenda Gray, Jo-Ann S. Passmore, Anna-Lise Williamson

**Affiliations:** 1 Institute of Infectious Disease and Molecular Medicine, University of Cape Town, Cape Town, South Africa; 2 Center for HIV and STIs, National Institute for Communicable Disease, National Health Laboratory Service, Johannesburg, South Africa; 3 Department of Pathology, Division of Medical Virology, University of Cape Town, Cape Town, South Africa; 4 UCT-MRC Clinical Gynaecological Cancer Research Centre, University of Cape Town, Cape Town, South Africa; 5 The South African Department of Science and Technology/National Research Foundation Centre of Excellence in Epidemiological Modelling and Analysis, Stellenbosch University, Stellenbosch, South Africa; 6 The Desmond Tutu HIV Centre, University of Cape Town, Cape Town, South Africa; 7 Seattle Children’s Research Institute, University of Washington, Seattle, United States; 8 Western Sydney Sexual Health Centre, Western Sydney Local Health District, Parramatta, Australia; 9 Marie Bashir Institute for Infectious Diseases and Biosecurity & Sydney Medical School-Westmead, University of Sydney, Sydney, Australia; 10 Perinatal HIV Research Unit, Faculty of Health Sciences, University of the Witwatersrand, Diepkloof, Johannesburg, South Africa; 11 Canada-African Prevention Trials Network, The Ottawa Hospital General Campus, Ottawa, Canada; 12 South African Medical Research Council, Cape Town, South Africa; 13 DST-NRF CAPRISA Centre of Excellence in HIV Prevention, University of Cape Town, Cape Town, South Africa; 14 National Health Laboratory Service, Groote Schuur Hospital, Observatory, Cape Town, South Africa; Rudjer Boskovic Institute, CROATIA

## Abstract

The objectives of the study were to investigate prevalence of cervical human papillomavirus (HPV) genotypes to inform HPV vaccination strategy in South Africa and to study factors associated with HPV prevalence. Sexually active, HIV-negative women, aged 16–22 years recruited from Soweto (n = 143) and Cape Town (n = 148) were tested for cervical HPV and other genital infections. Overall HPV prevalence was 66.7% (194/291) in young women. Cape Town women were more likely to have multiple HPV infections than the Soweto women (48.0%, 71/148 versus 35.0%, 50/143 respectively, p = 0.033) and probable HR-HPV types (34.5%, 51/148 versus 21.7%, 31/143 respectively, p = 0.022). The most frequently detected HPV types were HPV-16 (11.7%), HPV-58 (10.3%), HPV-51 (8.9%), HPV-66 (8.6%), HPV-18 and HPV-81 (7.6% each). HPV types targeted by the bivalent HPV vaccine (HPV-16/18) were detected in 18.6% (54/291) of women, while those in the quadrivalent vaccine (HPV-6/11/16/18) were detected in 24.7% (72/291) of women; and those in the nonavalent vaccine (HPV-6/11/16/18/31/33/45/52/58) were detected in 38.5% (112/291) of women. In a multivariable analysis, bacterial vaginosis remained significantly associated with HPV infection (OR: 4.0, 95% CI: 1.4–12.6). Women were more likely to be HPV positive if they had received treatment for STI during the past 6-months (OR: 3.4, 95% CI: 1.1–12.4) or if they had ever been pregnant (OR: 2.3, 95% CI: 1.1–5.5). Compared to women who reported only one sexual partner, those with increased number of lifetime sex partners were more likely to have HPV (4–10 partners: OR: 2.9, 95% CI: 1.1–8.0). The high prevalence of HPV types targeted by the nonavalent HPV vaccine encourages the introduction of this vaccine and catch-up HPV vaccination campaigns in South Africa. The high burden of BV and concurrent STIs also highlights the need to improve the prevention and appropriate management of sexually-acquired and other genital tract infections in South African youth.

## Introduction

Internationally, and in Africa, the majority of sexually transmitted infections (STIs) are in young people [1, 2]. Human papillomavirus (HPV) is one of the most common STIs and in women its prevalence peaks during adolescence, soon after sexual debut; and decreases with increasing age [3]. Those women who sexually debuted at ≤16 years are at higher risk for being HPV infected [3]. The median age of first sexual debut in South African women ranges from 16–18 years [4–6]. A high HPV prevalence in South African women <25 years of age has been previously reported in Gauteng and KwaZulu Natal Provinces (85% and 76% respectively) [7, 8]. In the Western Cape, estimates of HPV prevalence range from 44% to 71% [9–11].

HPV natural history is influenced by several factors, such as infection with other STIs, early sexual debut, increased number of lifetime sexual partners and increased numbers of current sexual partners [12–14]. In women, STIs and bacterial vaginosis (BV) are very prevalent in populations that are at high risk of human immunodeficiency virus (HIV) infection and among women between the ages of 15–44 years [15, 16]. BV, a dysbiosis rather than an STI, is thought to increase the risk of acquiring STIs [17].

There are currently three prophylactic HPV vaccines that are being rolled out internationally: Cervarix, Gardasil and Gardasil-9. Cervarix protects against two high-risk HPV types, HPV-16 and -18; Gardasil protects against two low-risk (LR) and two HR-HPV types, HPV-6, -11, -16 and -18; while Gardasil-9 protects against five more types in addition to those targeted by Gardasil, HPV-31, -33, -45, -52 and -58. Both Cervarix and Gardasil demonstrated cross protective efficacy against phylogenetically related HPV-16 and -18 types, such as HPV-31, -33, -45 and -51; however the duration of protection is not yet known. The efficacy, safety and immunogenicity of these vaccines have been recognized in well followed cohorts [18–20].

South Africa introduced a school-based national HPV vaccination program in 2014; at which time girls aged 9–10 years were vaccinated with Cervarix HPV vaccine and given in two doses at least six-months apart (to fit into the academic calendar year). The data on HPV genotype prevalence and distribution in unvaccinated populations is important to both inform vaccination campaigns as well as to monitor the impact on circulating HPV types after vaccination. As part of the HPV vaccination strategy in South Africa, it is important to have baseline data in adolescents and young women to assess the impact of vaccination and improve HPV vaccination strategies.

The primary objectives of this study were to investigate prevalence of cervical HPV infection in adolescent and young women, in particular the prevalence of HPV genotypes targeted by current HPV vaccines, and the pattern of co-infection with sexually transmissible pathogens [HPV, *Chlamydia trachomatis*, *Neisseria gonorrhoeae*, *Trichomonas vaginalis*, *Mycoplasma genitalium*, and herpes simplex virus-2 (HSV-2)]. The secondary objective was to study factors associated with HPV prevalence in this population.

## Materials and methods

### Study population and specimen collection

Between November 2013 and December 2014, 298 sexually experienced HIV-negative black women aged 16–22 years were recruited, all of whom were not vaccinated against HPV. Participants were enrolled from two disadvantaged urban African communities in Cape Town (South Peninsula) and Johannesburg (Soweto), South Africa, as part of the Women’s Initiative in Sexual Health (WISH) study [21] through community outreach programs and adolescent friendly sexual reproductive health services. The Human Research Ethics Committee of the University of Cape Town (HREC reference 267/2013) and University of the Witwatersrand (HREC reference M130745) approved the study. Written informed consent was obtained from participants who were ≥18 years while those who were 16–17 years old provided written assent and consent was obtained from their parent or guardian.

A HIV rapid test (Alere Determine™ HIV-1/2 Ag/Ab Combo, Alere, Waltham, MA) was performed. A lateral wall/posterior fornix swab was collected to determine vaginal pH and presence of BV. Endocervical samples were collected from each woman under speculum examination by clinicians using Digene cervical samplers (Digene Corporation Gaithersburg, MD, USA) and stored in Digene transport medium at -80°C until nucleic acid extraction.

### Detection of STIs, BV and vaginal pH

Nucleic acid was extracted using MagNA Pure Compact Nucleic Acid Isolation Kit (Roche). HPV genotyping was performed on extracted nucleic acid using the Roche Linear Array HPV genotyping test (Roche Molecular Systems, Pleasanton, CA, USA) which identifies 37 different HPV genotypes (HPV-6, -11, -16, -18, -26, -31, -33, -35, -39, -40, -42, -45, -51, -52, -53, -54, -55, -56, -58, -59, -61, -62, -64, -66, -67, -68, -69, -70, -71, -72, -73, -81, -82, -83, -84, -89 (HPV-CP6108) and–IS39) [22].

Discharge-causing organisms *C*. *trachomatis*, *N*. *gonorrhoeae*, *T*. *vaginalis*, *M*. *genitalium* and the ulcer-causing pathogens HSV and *Treponema pallidum* were diagnosed using two real-time multiplex PCRs on vulvovaginal swabs [23]. HSV-positive DNA extracts were re-tested and typed using a commercial HSV-1/HSV-2 PCR assay (Sacace Biotechnologies Srl, Como, Italy). *C*. *trachomatis* serovars L1-L3 were detected using a simplex real-time PCR as described by Morré et al. [24]. A lateral wall/posterior fornix swab was collected to determine vaginal pH and presence of BV. Nugent criteria were used to assess BV [25]. Participants with Nugent scores ≤3 were considered BV negative, 4–6 to have intermediate vaginal flora, while 7–10 were considered to have BV. Vaginal pH was measured using colour-fixed indicator strips (Macherey-Nagel, Düren Germany). Participants with STI symptoms were treated according to the syndromic management protocol. Those with STI positive laboratory results were contacted to attend for treatment.

### Statistical analyses

Data is first presented as combined for the two study sites, and further stratified by study sites because the participants from Cape Town were found to have significantly higher risk behaviour than those in Soweto [21]. Multiple HPV infections were defined as detection of two or more HPV types in the same sample. In cases where multiple infections were identified, individuals were counted as infected for the specific category if they have one or more infection in that category. However these women were counted more than once when determining the prevalence of LR-HPV, HR-HPV and probable HR-HPV if they have types that belong to more than one category.

“Logistic regression models assessed factors associated with baseline HPV prevalence. Multivariable models include factors that were simultaneously statistically significant at 5%, considering variables with p-value of less than 0.2 in the univariable analysis and manually selecting the final model based on both the effect size and p-value. In the multivariable analysis for HPV prevalence in both locations, the model was adjusted for location. Collinearity between factors was investigated and not found. Statistical analyses were performed using R”[26].

## Results

### HPV prevalence

**Fig 1:**
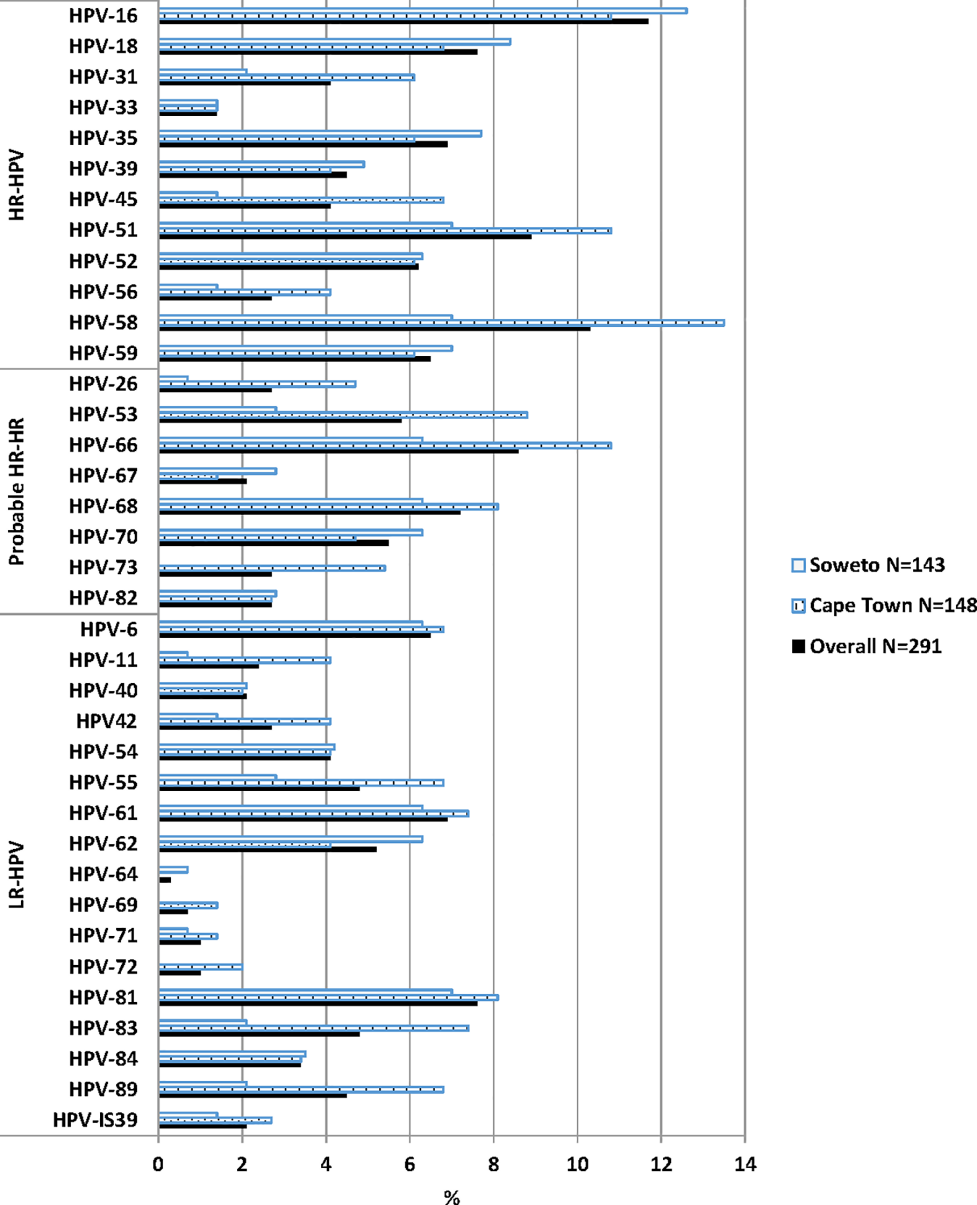
The prevalence of human papillomavirus genotypes according to study site in adolescents and young adults. HR-HPV types included HPV-16, -18, -31, -33, -35, -39, -45, -51, -52, -56, -58 and -59; probably or possible HR-HPV types included HPV-26, -53, -66, -67, -68, -70, -73 and -82; and low-risk (LR) HPV types HPV-6, -11, -40, 42, -54, -55, -61, -62, -64, -69, -71, -72, -81, -83, -84, -89 (HPV-CP6108) and–IS39 [22].

HPV data were available for 291 of the 298 women recruited. The overall HPV prevalence in these South African youth was 66.7% (194/291), being similarly prevalent in Cape Town and Soweto (68.2%, 101/148 versus 65.0%, 93/143; p = 0.648). Cape Town women were, however, more likely to have multiple HPV infections than the Soweto women (48.0%, 71/148 versus 35.0%, 50/143 respectively, p = 0.033) and probable HR-HPV types (34.5%, 51/148 versus 21.7%, 31/143 respectively, p = 0.022, Table 1). Overall, across the two sites, the most frequently detected HPV types were HPV-16 (11.7%), HPV-58 (10.3%), HPV-51 (8.9%), HPV-66 (8.6%), HPV-18 and HPV-81 (7.6% each). In Cape Town participants, the most prevalent HPV infections were HPV-58 (13.5%), HPV-16, HPV-51 and HPV-66 (10.8% each), HPV-53 (8.8%), HPV-68 and HPV-81 (8.1% each), HPV-61 and -83 (7.4% each). In the Soweto participants, HPV-16 (12.6%) was the most dominant type, followed by HPV-18 (8.4%), HPV-35 (7.7%), HPV-51, -58, -59 and 81 (7.0% each, Fig 1).

**Table 1:** Prevalence of any human papillomavirus (HPV), multiple infections, single infection, HR-HPV, probable HR-HPV and LR-HPV.

	OVERALL N = 291	CAPE TOWN, N = 148	SOWETO, N = 143	p-value*
	n, % (95% CI)	n, % (95% CI)	n, % (95% CI)	
**Any HPV**	194, 66.7% (61.3–72.1%)	101, 68.2% (60.7–75.7%)	93, 65.0% (57.2–72.8%)	0.648
**Multiple HPV infections**	121, 41.6% (35.9–47.3%)	71, 48.0% (40.0–56.0%)	50, 35.0% (27.2–42.8%)	**0.033**
**Single HPV infection**	73, 25.1% (20.1–30.1%)	30, 20.3% (13.8–26.8%)	43, 30.1% (22.6–37.6%)	0.073
**HR-HPV types**	133, 45.7% (40.0–51.4%)	66, 44.6% (36.6–52.6%)	67, 46.9% (38.7–55.1%)	0.788
**Probable HR-HPV types**	82, 28.2% (23.0–33.4%)	51, 34.5% (26.8–42.2%)	31, 21.7% (14.9–28.5%)	**0.022**
**LR-HPV types**	112, 38.5% (32.9–44.1%)	64, 43.2% (35.2–51.2%)	48, 33.6% (25.9–41.3%)	0.115

* p-value of chi-square test to compare frequencies from Cape Town and Soweto sites.

HR-HPV: HPV-16, -18, -31, -33, -35, -39, -45, -51, -52, -56, -58 and -59. Probable HR-HPV: HPV-26, -53, -66, -67, -68, -70, -73 and -82. LR-HPV: HPV-6, -11, -40, 42, -54, -55, -61, -62, -64, -69, -71, -72, -81, -83, -84, -89 (HPV-CP6108) and–IS39

### Prevalence of HPV types targeted by bivalent, quadrivalent and nonavalent HPV vaccines

**Fig 2:**
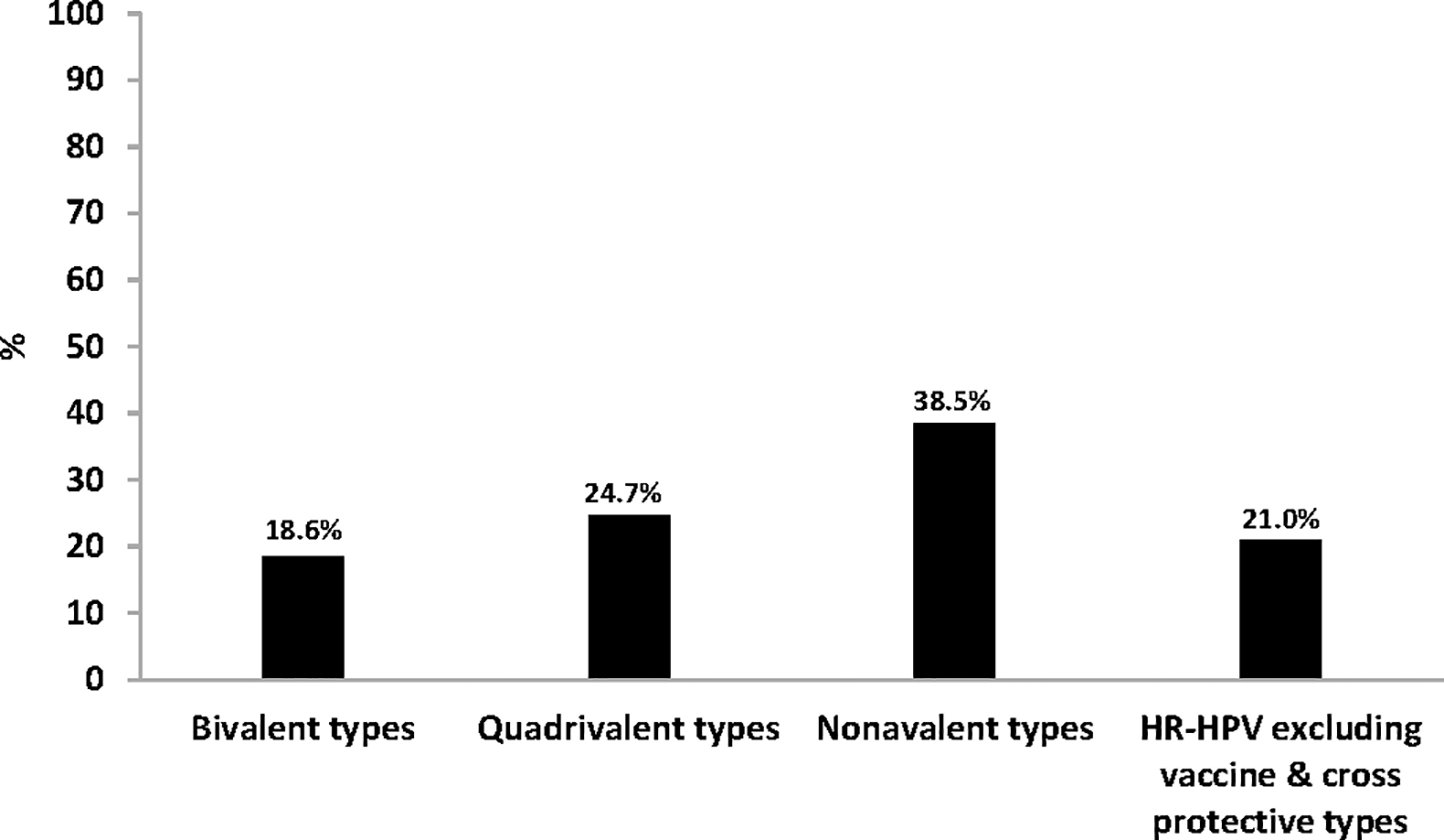
Prevalence of human papillomavirus (HPV) types targeted by current vaccines and non-vaccine types. Bivalent types: one or both HPV types targeted by the Cervarix HPV vaccine (HPV16/18); Quadrivalent types: one or more HPV types targeted by the Gardasil HPV vaccine (HPV6/11/16/18); Nonavalent types: one or more HPV types targeted by the Gardasil-9 HPV vaccine (HPV6/11/16/18/31/33/45/52/58); HR-HPV excluding vaccine & cross protective types (HPV-35/39/56/59).

HPV types targeted by the bivalent HPV vaccine (HPV-16/18) were detected in 18.6% (54/291) of women overall, while those found in the quadrivalent vaccine (HPV-6/11/16/18) were detected in 24.7% (72/291) of women; and those in the nonavalent vaccine (HPV-6/11/16/18/31/33/45/52/58) were detected in 38.5% (112/291) of women (Fig 2). Presently South Africa is vaccinating with the Bivalent vaccine. HR-HPV types that are not targeted by bivalent, quadrivalent or nonavalent HPV vaccines were observed in 21.0% (61/291 of the women (HPV-35/39/56/59, Fig 2).

### Prevalence and pattern of HPV co-infection with other STIs

**Fig 3:**
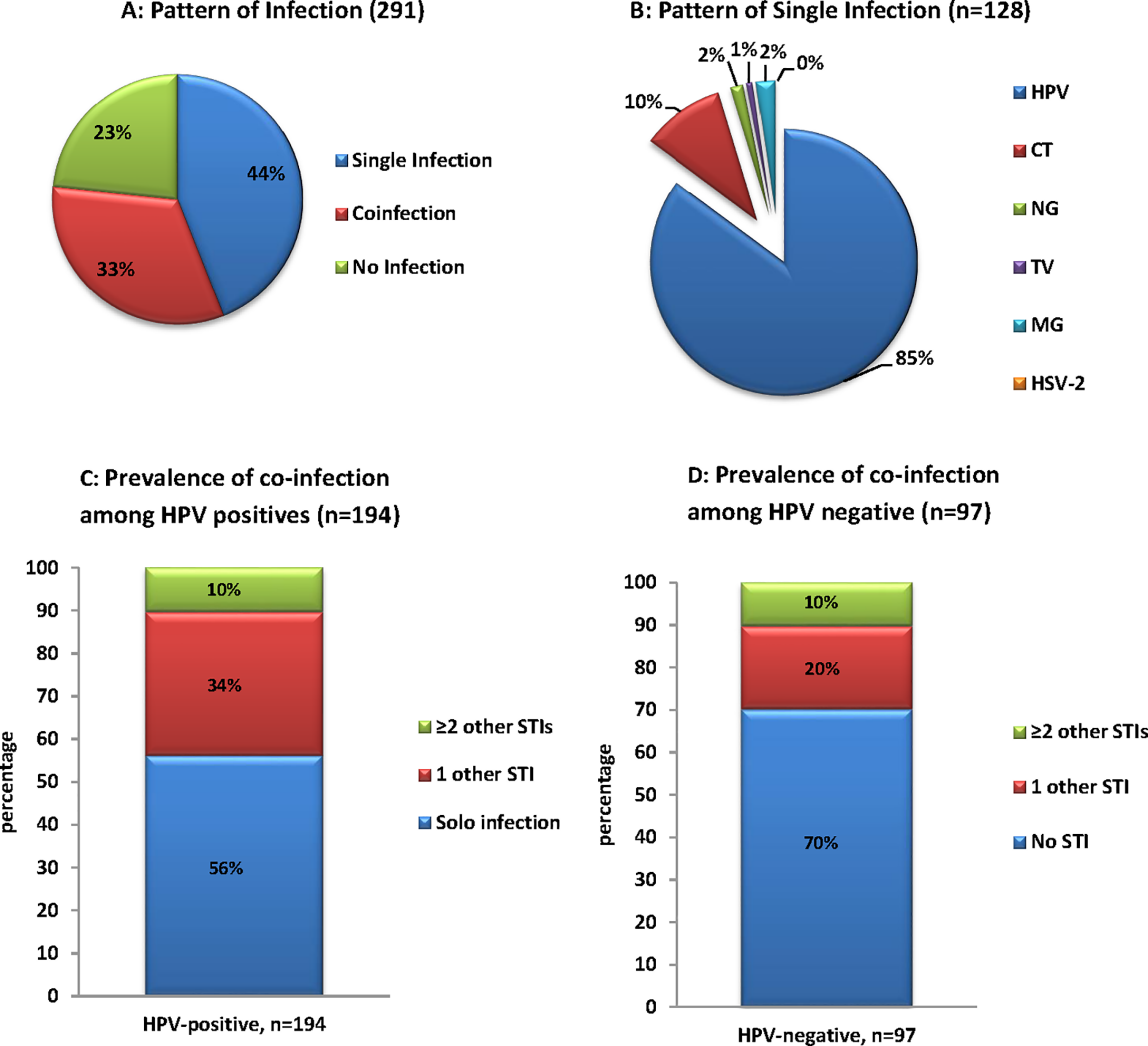
Pattern of sexual transmitted infection among South African adolescents and young women (A) Pattern of single infection (HPV or CT or NG or TV or MG or HSV-2), co-infection with more than one of the six STIs; (B) Pattern of infection among women with one of the six investigated STIs; (C) Prevalence of co-infections^a^ among South African adolescents and young women with one or more sexual transmitted infections. ^a^ among 194 women that were HPV positive, 56% were positive for HPV only (Solo infection); 34% were co- infected with CT or NG or TV or MG or HSV-2 and 10% were infected with ≥2 other STI; (D) Prevalence of co-infections^b^ among South African adolescents and young women with one or more sexual transmitted infections. ^b^ among 97 women that were HPV negative, 70% were negative for all tested STIs, 20% were infected with CT or NG or TV or MG or HSV-2 and 10% were infected with ≥2 other STI. HPV: Human papillomavirus. CT: *Chamydia trachomatis*. NG: *Neisseria gonorrhoeae*. TV: *Trichomanas vaginalis*. MG: *Mycoplasma genitalium*. HSV-2: Herpes simplex virus-2.

HPV was the most prevalent STI detected (66.7% 194/291) in these adolescent and young women. Co-infection with another STIs (including *C*. *trachomatis*, *N*. *gonorrhoeae*, *T*. *vaginalis*, *M*. *genitalium* or HSV-2) was observed in 44.0% (128/291) women while co-infection with ≥2 STIs was observed in 32.6% (95/291, Fig 3A). When investigating the pattern of infection among women who presented with single STI infection, HPV was most prevalent (85.2%, 109/128) followed by *C*. *trachomatis* (10.2%, 13/128, Fig 3B). Among 194 women that were HPV positive, 56.2% (109/194) were infected with only HPV; 33.5% (65/194) were co-infected with another STI (*C*. *trachomatis*, *N*. *gonorrhoeae*, *T*. *vaginalis*, *M*. *genitalium* or HSV-2) and 10.3% (20/194) were infected with ≥2 other STIs (Fig 3C). Among 97 women that were HPV negative, 70.1% (68/97) were not infected with any investigated STIs; 19.6% (19/97) were infected with one STI (*C*. *trachomatis*, *N*. *gonorrhoeae*, *T*. *vaginalis*, *M*. *genitalium* or HSV-2) and 10.3% (10/97) were infected with ≥2 other STIs (Fig 3D). When further investigating women that were infected with HPV and one other STI; HPV together with *C*. *trachomatis* was the most common co-infection observed (70.8%, 46/65) followed by HPV/*T*. *vaginalis* co-infection (12.3%, 8/65); HPV/*N*. *gonorrhoeae* co-infection (7.7%, 5/65); HPV/HSV-2 co-infection (4.6%, 3/65) and HPV/*M*. *genitalium* (4.6%, 3/65).

### Factors associated with HPV prevalence in adolescents and young women

Overall, women with BV (Nugent score ≥7) and those with intermediate vaginal flora (Nugent 4–6) were more likely to be HPV positive than women who were BV negative (Nugent ≤3) [odds ratio (OR): 2.8, 95% confidence interval (CI): 1.3–6.8; OR: 2.2, 95% CI: 1.3–3.7, respectively]. Having a raised vaginal pH (>4.5) was not significantly associated with HPV infection in women (OR: 1.7, 95% CI: 0.9–2.9). Women infected with any other STI were more likely to have HPV infection than women with no STI (OR: 1.9, 95% CI: 1.2–3.3). Women who visited medical care facility to receive family planning (OR 1.8, 95% CI 1.0–3.4) or who had ever been pregnant (OR 2.3, 95% CI 1.1–5.5) were more likely to be HPV positive than those who did not (Table 2). Increased numbers of lifetime sexual partners was positively associated with prevalent HPV (4–10 partners: OR 3.0, 95% CI 1.2–7.6); however, the age of sexual debut was not associated with HPV. Women who reported a preference for male condom as their primary form of contraception were less likely to have HPV infection than those who preferred other methods (OR 0.5, 95% CI 0.3–1.0), although the frequency of reported male condom use was not associated with prevalent HPV (Table 2).

**Table 2:** Human papillomavirus prevalence and factors associated with human papillomavirus in adolescents and young women (univariate analysis).

		OVERALL			CAPE TOWN			SOWETO	
Variable	HPV prevalence	OR (95% CI)	p-value	HPV prevalence	OR (95% CI)	p-value	HPV prevalence	OR (95% CI)	p-value
**Age**		1.0 (0.9–1.2)	0.897		0.8 (0.7–1.0)	0.110		1.2 (1.0–1.5)	0.117
**Bacterial Vaginosis**									
negative	62/113 (55%)	ref		37/61 (61%)	ref		25/52 (48%)	ref	
intermediate	95/131 (73%)	2.2 (1.3–3.7)	**0.004**	51/70 (73%)	1.7 (0.8–3.7)	0.140	44/61 (72%)	2.8 (1.3–6.2)	**0.010**
positive	31/40 (78%)	2.8 (1.3–6.8)	**0.014**	13/17 (77%)	2.1 (0.7–8.2)	0.236	18/23 (78%)	3.9 (1.3–13.2)	**0.019**
***Chlamydia trachomatis***		** **			** **	** **	** **	** **	** **
negative	128/201 (63.7%)	ref		57/86 (66%)	ref		71/115 (62%)	ref	
positive	64/87 (73.6%)	1.6 (0.9–2.8)	0.104	44/62 (71%)	1.2 (0.6–2.6)	0.546	20/25 (80%)	2.5 (0.9–7.9)	0.090
***Neisseria gonorrhoeae***									
negative	175/264 (66%)	Ref		90/131 (69%)	ref		85/133 (64%)	ref	
positive	17/24 (70.8%)	1.2 (0.5–3.3)	0.652	11/17 (65%)	0.8 (0.3–2.6)	0.739	6/7 (86%)	3.4 (0.6–65)	0.265
***Trichomonas vaginalis***									
negative	181/272 (67%)	ref		93/137 (68%)	ref		88/135 (65%)	ref	
positive	11/16 (69%)	1.1 (0.4–3.6)	0.856	8/11 (73%)	1.3 (0.4–6.0)	0.740	3/5 (60%)	0.8 (0.1–6.2)	0.812
***Mycoplasma genitalium***									
negative	185/278 (67%)	ref		97/142 (68%)	ref		88/136 (65%)	ref	
positive	7/10 (70%)	1.2 (0.3–5.5)	0.820	4/6 (67%)	0.9 (0.2–6.9)	0.933	3/4 (75%)	1.6 (0.2–33.6)	0.673
**Herpes simplex virus-2**									
negative	185/280 (66%)	ref		95/141 (67%)	ref		90/139 (65%)	…	
positive	7/8 (88%)	3.6 (0.6–67.7)	0.235	6/7 (86%)	2.9 (0.5–55.7)	0.330	1/1 (100%)	…	0.987
**Any STI**									
negative	107/175 (61%)	ref		44/71 (62%)	ref		63/104 (61%)	ref	
positive	85/113 (75%)	1.9 (1.2–3.3)	**0.014**	57/77 (74%)	1.8 (0.9–3.6)	0.117	28/36 (78%)	2.3 (1.0–5.8)	0.066
**Vaginal pH**									
≤4.5	43/74 (58%)	ref		26/44 (59%)	ref		17/30 (57%)	ref	
>4.5	137/197 (70%)	1.7 (0.9–2.9)	0.077	68/95 (72%)	1.7 (0.8–3.7)	0.145	69/102 (68%)	1.6 (0.7–3.7)	0.269
**Received treatment of STIs in past 6 months**									
No	119/181 (66%)	ref		69/102 (68%)	ref		50/79 (63%)	ref	
Yes	22/26 (85%)	2.9 (1.0–10.1)	0.063	12/14 (86%)	2.9 (0.7–19.1)	0.183	10/12 (83%)	2.9 (0.7–19.7)	0.188
**Family planning received past 6 months**									
No	44/74 (60%)	ref		13/21 (62%)	ref		31/53 (59%)	ref	
Yes	97/133 (73%)	1.8 (1.0–3.4)	**0.047**	68/95 (72%)	1.6 (0.6–4.1)	0.384	29/38 (76%)	2.3 (0.9–6.0)	0.080
**HIV testing received past 6 months**									
No	50/81 (62%)	ref		18/25 (72%)	ref		32/56 (57%)	ref	
Yes	91/126 (72%)	1.6 (0.9–2.9)	0.115	63/91 (69%)	0.9 (0.3–2.3)	0.789	28/35 (80%)	3.0 (1.2–8.5)	**0.028**
**Ever pregnant**									** **
No	95/150 (63%)	ref		58/86 (67%)	ref		37/64 (58%)	ref	** **
Yes	46/57 (81%)	2.4 (1.2–5.3)	**0.019**	23/30 (77%)	1.6 (0.6–4.4)	0.346	23/27 (85%)	4.2 (1.4–15.6)	**0.016**
**Prefer male condom for contraception**									** **
No	113/157 (72%)	ref		73/103 (71%)	ref		40/54 (74%)	ref	** **
Yes	28/50 (56%)	0.5 (0.3–1.0)	**0.037**	8/13 (62%)	0.7 (0.2–2.3)	0.492	20/37 (54%)	0.4 (0.2–1.0)	0.050
**Sexual partner/s HIV positive (Discordancy)**									** **
No	117/164 (71%)	ref		73/105 (70%)	ref		44/59 (75%)	ref	** **
Yes	24/43 (56%)	0.5 (0.3–1.0)	0.054	8/11 (73%)	1.2 (0.3–5.6)	0.826	16/32 (50%)	0.3 (0.1–0.8)	**0.020**
**Age at first sex**									
12–15 years	48/64 (75%)	ref		31/38 (82%)	ref		17/26 (65%)	ref	
16–17 years	72/114 (63%)	0.6 (0.3–1.1)	0.108	40/64 (63%)	0.4 (0.1–1.0)	0.047	32/50 (64%)	0.9 (0.3–2.5)	0.905
18–20 years	13/18 (72%)	0.9 (0.3–3.0)	0.812	4/6 (67%)	0.5 (0.1–3.7)	0.409	9/12 (75%)	1.6 (0.4–8.5)	0.555
**Number of lifetime sexual partners**									
1	32/58 (55%)	ref		15/22 (68%)	ref		17/36 (47%)	ref	
2–3	71/101 (70%)	1.9 (1.0–3.8)	0.056	44/65 (68%)	1.0 (0.3–2.7)	0.966	27/36 (75%)	3.4 (1.3–9.4)	**0.018**
4–10	33/42 (79%)	3.0 (1.2–7.6)	**0.018**	18/24 (75%)	1.4 (0.4–5.2)	0.609	15/18 (83%)	5.6 (1.5–27.2)	**0.016**

BV: bacterial vaginosis. BV Negative: nugent score <4. BV inter: nugent score 4–6. BV Positives: nugent score ≥7. OR: odds ratio. CI: confidence interval.

In a multivariable analysis, BV remained significantly associated with HPV infection (OR: 4.0, 95% CI: 1.4–12.6). Women were more likely to be HPV positive if they had received treatment for STI during the past 6-months (OR: 3.4, 95% CI: 1.1–12.4) or if they had ever been pregnant (OR: 2.3, 95% CI: 1.1–5.5). Compared to women who reported only one sexual partner, those with increased number of lifetime sex partners were more likely to have HPV (4–10 partners: OR: 2.9, 95% CI: 1.1–8.0; Table 3).

**Table 3:** Human papillomavirus prevalence and factors associated with human papillomavirus in adolescents and young women (multivariable analysis).

Variable	aOR (95% CI)	p-value
**OVERALL PARTICIPANTS**		
**Bacterial Vaginosis**		
negative	ref	
intermediate	2.6 (1.3–5.2)	**0.007**
positive	4.0 (1.4–12.6)	**0.012**
**Received treatment of STIs in past 6 months**		
No	ref	
Yes	3.4 (1.1–12.4)	**0.042**
**Ever pregnant**		
No	ref	
Yes	2.3 (1.1–5.5)	**0.040**
**Number of lifetime sexual partners**		
1	ref	
2–3	1.9 (0.9–4.0)	0.110
4–10	2.9 (1.1–8.0)	**0.036**
**SOWETO PARTICIPANTS**		
**Bacterial Vaginosis**		
negative	ref	
intermediate	4.7 (1.5–16.6)	**0.011**
positive	7.2 (1.4–45.2)	**0.020**
**Sexual partner/s HIV positive (Discordancy)**		
No	ref	
Yes	0.2 (0.04–0.5)	**0.003**
**Number of lifetime sexual partners**		
1	ref	
2–3	5.9 (1.8–22.0)	**0.005**
4–10	9.3 (2.0–57.7)	**0.008**

When young women were grouped according to study site, those from Soweto with BV (Nugent score ≥7) and those with intermediate BV (Nugent 4–6) were more likely to be HPV positive than women who were BV negative (Nugent ≤3, OR: 3.9, 95% CI: 1.3–13.2; OR: 2.8, 95% CI: 1.3–6.2, respectively). Soweto women who visited a medical care facility to receive an HIV test during the past 6-months (OR 3.0, 95% CI 1.2–8.5) or those who had ever been pregnant (OR 4.2, 95% CI 1.4–15.6) were more likely to be HPV positive. Reported increased numbers of lifetime sex partners in Soweto was positively associated with HPV prevalence (2–3 partners: OR 3.4, 95% CI 1.3–9.4 and 4–10 partners: OR 5.6, 95% CI 1.5–27.2). In addition, Sowetan women who reported HIV-positive sexual partners were less likely to have HPV infection than those who reported only HIV-negative partners or partners of unknown HIV status (OR 0.3, 95% CI 0.1–0.8, Table 2).

In a multivariable analysis including only Sowetan participants, BV remained significantly associated with HPV infection (OR 7.2, 95% CI 1.5–45.2). Sowetan women who reported only one sexual partner, women with an increased numbers of lifetime sex partners were more likely to have HPV (2–3 partners: OR 5.9, 95% CI 1.8–22.0; 4–10 partners: OR 9.3, 95% CI 2.0–57.7). Sowetan women who reported sex with HIV-positive partners were less likely to have HPV infections than those reporting HIV negative partners or partners of unknown HIV status (OR 0.2, 95% CI 0.0–0.5; Table 3). Among Cape Town girls, only age at first sex was related to HPV positivity. In contrast to women from Soweto, there was no significant association between HPV prevalence and other factors for participants from Cape Town.

## Discussion

We have described HPV prevalence among adolescents and young women from two Provinces of South Africa. Among these HIV-negative young women, the high overall HPV prevalence (66.7%) and high HPV-16 prevalence (11.7%) is of substantial public health concern. HPV-16 is the dominant HPV type in cervical cancer cases occurring in more than 50% of cervical cancers [12]. Furthermore, HPV-16 is also the most carcinogenic HPV and more likely to persist than other HPV types. After 3–5 years of persistent infection, HPV-16 is reported to have a risk of a pre-cancer diagnosis of nearly 40% [27].

A high HPV prevalence among adolescents and young women has previously been reported in Gauteng, KwaZulu Natal and Western Cape Provinces of South Africa [8–11]. There was no difference in the prevalence of HPV infection between Cape Town (Western Cape) and Soweto (Gauteng) in this study. The high HPV prevalence seen in this population of HIV-negative adolescents and young women puts them at increased risk of HIV acquisition [9, 28]. The association between HIV and HPV is well documented, with HPV increasing the risk of HIV acquisition and in turn HIV increasing the risk of HPV acquisition and persistence [29, 30]. Even though adolescents are at low risk of cervical cancer, HIV-positive adolescents are at high risk for abnormal cervical cytology and are more likely to have persistent HPV infections [11, 31].

We acknowledge that HPV types targeted by vaccines should be studied in cervical cancer studies and that the HPV prevalence in a young population with no cervical cancer does not reflect the proportion of types found in cancers. However, the high prevalence (38.5%) of HPV types targeted by the Gardasil-9 HPV vaccine in this population encourages the introduction of Gardasil-9. This vaccine targets seven HPV types that cause cancer, and two HPV types that cause genital warts. In South Africa, coverage of more than 90% was achieved in 2014 when a national school based HPV vaccination programme was introduced in public schools for girls of 9–10 years of age [32]. Our results illustrate the high prevalence of HPV in young women and support the continuation of large scale roll-out of HPV vaccination to South African girls and the establishment of catch-up campaigns for young adolescents.

The current HPV vaccines are prophylactic and are not effective in women who are already infected with HPV; therefore continuation of cervical cancer screening programs is essential in order to reduce cervical cancer through early detection of cervical precancerous lesions and treatment among women who are already HPV infected, unvaccinated or partially vaccinated women, and those infected with HPV types not targeted by current HPV vaccines. The high prevalence of HR-HPV types that are not targeted by current vaccines (23.4%), specifically HPV-51 and HPV-35, also raises concern in this population. HPV 51 and 35 have been reported in 2.1% and 9.7% of African cervical cancers respectively [33].

A high burden of concurrent STIs was observed in this study, similar to a recently reported study among Western Cape young women [34]. In addition to HPV, other STIs increase the risk of HIV acquisition and transmission [35]. Here, we also found an association between BV and HPV. BV has been previously associated with prevalent or new HPV infections and low grade squamous intraepithelial lesions [36, 37]. However, women with high vaginal pH were not more likely to be HPV infected than women with low vaginal pH; implying the mechanisms of BV-induced barrier disruption may be independent of pH. Instead, inflammation induced by specific vaginal microbiota may compromise barrier integrity [38]. It has been also shown that specific bacteria associated with BV such as *Gardnerella vaginalis* are associated with host-epithelium disruption and that soluble products from these bacteria inhibited wound healing. It is therefore feasible that the same proposed mechanism that increased the risk of HIV infection could also result in increased HPV infection in women with BV [39]. Women that do not have BV but have a vaginal microbiota dominated by *Lactobacillus crispatus* have lower prevalent HPV implying that *L*. *crispatus* may provide protection from HPV infection whereas greater microbiota diversity is associated with cervical intraepithelial neoplasia disease progression [40, 41]. Among Soweto participants there was an association between BV with HPV but not among Cape Town participants. This could be due to the high prevalence of HPV (61%) in BV negative Cape Town participants compared to Sowetan where 48% of the BV negative women were HPV positive.

As expected, sexual behaviour had an impact on HPV prevalence. Increased number of lifetime sexual partners, pregnancy, infection with other STIs and prior treatment for STIs were associated with HPV prevalence in multivariable analysis. Increased number of pregnancies and early maternal age at first birth were associated with increased risk of cervical cancer [42]. HPV infection was less likely among women who reported having an HIV-positive sexual partner than those with HIV-negative or unknown partner’s HIV status. These findings could reflect increased frequency of condom usage among this group [43].

It is important to note that current study participants we recruited from sexual reproductive health services and community outreach programs. According to second South African National Youth Risk Behaviour Survey conducted in nine Provinces of South Africa; 41% of sexual active participants reported to have had more than 2 sexual partners in lifetime; 4.4% reported ever having had STI and 19.2% had been pregnant. In contrast, 71.1% (57/207) of current study participants reported to have had more than 2 lifetime sexual partners; 15.0% (36/207) reported ever having STI and 27.5% to have been pregnant. Therefore; current study participants have high risk sexual behaviour compared to general population.

## Conclusion

This study observed high HPV prevalence in adolescents and young HIV-negative women. The high prevalence of HPV types targeted by current HPV vaccines especially HPV-16, suggests that young South African women would greatly benefit from HPV vaccination. The high prevalence of HPV types targeted by Gardasil-9 HPV vaccine supports the introduction of Gardasil-9 HPV vaccine as this vaccine targets larger number of HR-HPV types that cause cancer. These findings also encourage the continuation of large scale roll-out of HPV vaccination and catch-up programs in South Africa. The findings of this study could help inform health policy makers by providing useful HPV baseline data for assessing the impact of HPV vaccination in these communities, and possibly assist in development of policy to improve HPV vaccination strategies. The high burden of BV and concurrent STIs also highlights the urgent need to improve the prevention, detection and appropriate management of sexually-acquired and other genital tract infections in adolescents and young women in South Africa.
